# 2345. Factors associated with COVID-19 vaccination refusal among U.S. Immigration and Customs Enforcement detainees

**DOI:** 10.1093/ofid/ofad500.1967

**Published:** 2023-11-27

**Authors:** Carlo Foppiano Palacios, Mark Travassos

**Affiliations:** Yale University, New Haven, Connecticut; University of Maryland School of Medicine, Baltimore, Maryland

## Abstract

**Background:**

COVID-19 has disproportionately affected migrant detainees, leading to SARS-CoV-2 test positivity rates exceeding 50% among migrants in U.S. Immigration and Customs Enforcement (ICE) detainment facilities. The federal government started vaccinating migrants in detention centers in January 2021 for SARS-CoV-2, but little data on vaccine uptake is available. We sought to identify ICE facility characteristics and particular vaccines associated with vaccination refusal in migrant detention centers.

**Methods:**

As result of Freedom of Information Act request, we acquired a comprehensive dataset of SARS-CoV-2 immunization efforts across ICE facilities from January, 2021 to July, 2022. Data included number of vaccines offered and refused, brand of vaccine (Janssen, Moderna, or Pfizer), vaccination date, detention facility name, and whether ICE Health Service Corps (IHSC) staffed the vaccine delivery. We incorporated data from ICE website on each detention center’s ownership, management of operations, detainee capacity, and COVID-19 burden (as of January 11th, 2021). Descriptive statistics, chi-square, Welch’s ANOVA, and multivariate logistic regression were performed.

**Results:**

Of 129,046 vaccines offered, 65,015 (50.4%) were refused across 103 detention centers across 29 states on 506 vaccination dates. Factors associated with higher refusal included facilities not operated by IHSC (p< 0.001), local government operation (p=0.03), private operations (p=0.02), and vaccinations during the 2^nd^ (p< 0.001) and 3^rd^ quarters (p< 0.001) of 2022. Factors associated with lower refusal included ownership by local or state government, other public association, or private company; combination private/public operations; any COVID-19 death within ICE facility; and vaccination with Moderna and Pfizer (all p< 0.005).

**Figure 1.**
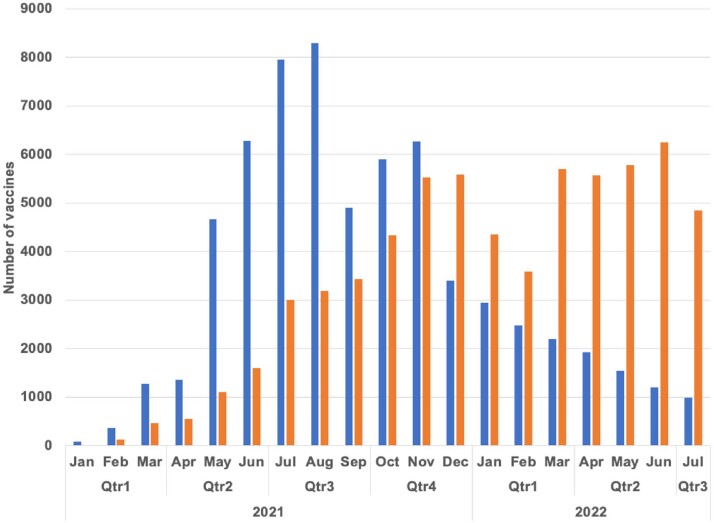
Numbers of SARS-CoV-2 vaccinations administered (blue) and vaccine refusals (orange) in United States Immigration and Customs Enforcement facilities.

**Conclusion:**

Half of ICE detainees refused vaccination against SARS-CoV-2. Refusal rates were lower among vaccination campaigns utilizing mRNA vaccines and among facilities with documented COVID-19 death. Addressing refusal rates may involve detainee education on the efficacy and safety of different SARS-CoV-2 vaccines.

**Disclosures:**

**All Authors**: No reported disclosures

